# NATIONAL POST–ACUTE AND LONG-TERM CARE STUDY: VARIATION IN OPERATING STATUS OF ADULT DAY CENTERS DUE TO COVID-19

**DOI:** 10.1093/geroni/igad104.0104

**Published:** 2023-12-21

**Authors:** Jessica Lendon, Priyanka Singh, Zhaohui Lu, Christine Caffrey, Manisha Sengupta

**Affiliations:** CDC National Center for Health Statistics, Hyattsville, Maryland, United States; National Center for Health Statistics, Hyattsville, Maryland, United States; National Center for Health Statistics, Hyattsville, Maryland, United States; National Center for Health Statistics, Hyattsville, Maryland, United States; National Center for Health Statistics, Hyattsville, Maryland, United States

## Abstract

The 2020 and 2022 National Post-acute and Long-term Care Study (NPALS) captured nationally representative data on ADSCs’ operating statuses to mitigate COVID-19 among participants and staff. In 2020/2021, 35% of ADSCs were physically open and only served participants onsite, 32% were physically closed but serving participants at places of residence (i.e. virtually, meal delivery, home-visits), 20% served participants onsite and at places of residence, and 13% were temporarily closed and not serving participants. There was statistically significant variation in operating status by region, policy-relevant organizational and participant-level characteristics, and in the types of services provided. Discussion of results will highlight how NPALS data can be used to assess disparities in ADSC availability during the pandemic. Additional information from the forthcoming 2022 survey data will also be discussed, focusing on potential differences in operating status and ADSC characteristics between the two survey waves.

